# Autophagy Modulation as a Potential Therapeutic Strategy in Osteosarcoma: Current Insights and Future Perspectives

**DOI:** 10.3390/ijms241813827

**Published:** 2023-09-07

**Authors:** Sergio Almansa-Gómez, Francisco Prieto-Ruiz, José Cansado, Marisa Madrid

**Affiliations:** Yeast Physiology Group, Departamento de Genética y Microbiología, Facultad de Biología, Universidad de Murcia, 30100 Murcia, Spain; sergio.almansag@um.es (S.A.-G.); francisco.prieto@um.es (F.P.-R.)

**Keywords:** autophagy, cancer, modulation, osteosarcoma, chemoresistance, radioresistance, treatment

## Abstract

Autophagy, the process that enables the recycling and degradation of cellular components, is essential for homeostasis, which occurs in response to various types of stress. Autophagy plays an important role in the genesis and evolution of osteosarcoma (OS). The conventional treatment of OS has limitations and is not always effective at controlling the disease. Therefore, numerous researchers have analyzed how controlling autophagy could be used as a treatment or strategy to reverse resistance to therapy in OS. They highlight how the inhibition of autophagy improves the efficacy of chemotherapeutic treatments and how the promotion of autophagy could prove positive in OS therapy. The modulation of autophagy can also be directed against OS stem cells, improving treatment efficacy and preventing cancer recurrence. Despite promising findings, future studies are needed to elucidate the molecular mechanisms of autophagy and its relationship to OS, as well as the mechanisms underlying the functioning of autophagic modulators. Careful evaluation is required as autophagy modulation may have adverse effects on normal cells, and the optimization of autophagic modulators for use as drugs in OS is imperative.

## 1. Introduction

Osteosarcoma (OS), the most common type of malignant bone tumor in adolescents and children, can occur at any age [1]. The incidence rate is estimated to be 2 to 4 per million per year, with a slightly higher male incidence [2]. It is very heterogeneous in origin and presentation, although its occurrence in the metaphyses of long bones suggests that it involves rapid bone production or turnover [3].

Clinically, OS is often treated using a combined approach, including neoadjuvant chemotherapy with doxorubicin (DOX), cisplatin (CDDP), methotrexate (MTX), ifosfamide (IFO), and surgery. This strategy has significantly improved survival in patients (from <20% to 55–70% at five years), although there are still problems relating to side effects or relapse. The causes of chemoresistance in OS cells include increased levels of autophagy under various types of stress, including mainly metabolic and therapeutic [4]. Due to tumor radioresistance, radiotherapy is rarely used to treat OS [3]. Despite these advances, overall survival has reached a bottleneck [5]. Since no significant advances in treating the disease have been made during recent years, the search for new therapeutic strategies is justified, where autophagy has become an exciting option due to its relationship with multiple biological processes in OS. In this review, we underscore the significance of understanding autophagy in the context of OS pathophysiology, highlighting the potential of autophagy modulation as a future therapeutic avenue.

## 2. Autophagy

Autophagy is a cellular catabolic process that supplies energy and metabolites by recycling and degrading cellular components through the lysosome (in higher eukaryotes) or vacuoles (in lower eukaryotes) [6]. Autophagy is constitutively active at low levels and acts as a quality control mechanism, degrading damaged organelles, misfolded/misplaced proteins, enzyme complexes, and other damaged or unnecessary cellular structures [7]. In addition, this process is induced at a high intensity under stress conditions and, through the recycling of cellular components, directly contributes to cellular metabolism by providing building blocks and energy [8].

Autophagy is involved in several physiological processes, including immunity, development, differentiation, metabolism, and even cell death [7,9,10]. In fact, autophagy and apoptosis overlap along multiple pathways. However, altered autophagy regulation has been associated with numerous pathological conditions. These include myopathies, heart disease, various neurodegenerative diseases, and cancer [11]. In the latter, it plays a dual role depending on the cellular context, even promoting or suppressing carcinogenesis [12]. Its function depends on many factors, such as the type of tumor, its stage, or the characteristics of the host, and must be considered in a complex scenario [13]. Despite these difficulties, autophagy is currently under investigation as a future therapeutic target for many cancers [11].

In mammals, it is possible to identify three different types of autophagy: macroautophagy, microautophagy, and chaperone-mediated autophagy (CMA). Despite their differences, all promote the degradation of cytosolic components in the lysosome [14]. This review focuses specifically on macroautophagy: the primary and more extensively studied type of autophagy in cells. Therefore, we refer to it as autophagy unless otherwise stated.

### 2.1. Autophagy Molecular Machinery and Regulation

**Figure 1:**
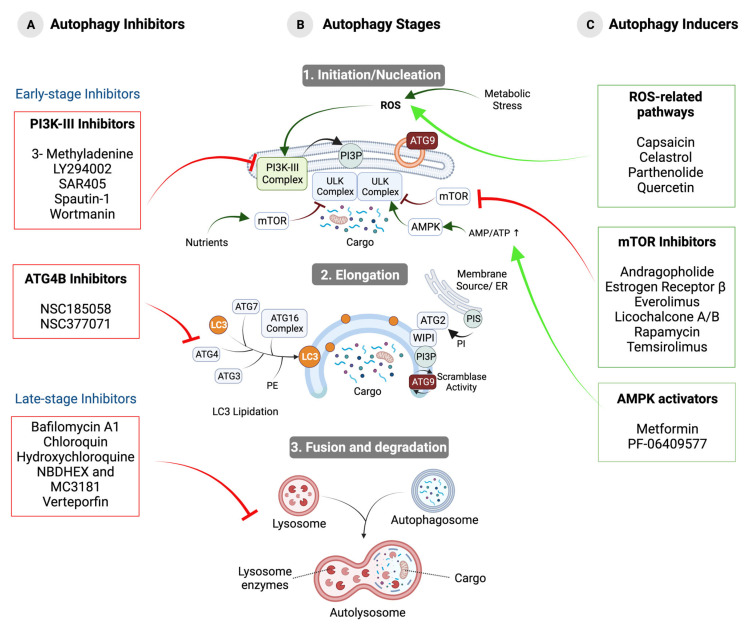
Stages of autophagy and the effect of modulating compounds on the progression of this process. (**A**) Autophagy inhibitors. Early-stage inhibitors that affect autophagosome formation and inhibit PI3K-III and ATG4B and late-stage inhibitors that affect lysosomal function by interfering with autophagosome–lysosome fusion or cargo degradation are shown. (**B**) Stages of autophagy. (1) Initiation/nucleation: under stress conditions, IM synthesis is initiated around the cargo by the recruitment and activation of ULK Complex, PI3K-III Complex, and ATG9 vesicles. (2) Elongation: expansion of the IM by membrane lipid addition driven by the ATG2–WIPI complex, ATG9, and LC3 lipidation. (3) Fusion and degradation: fusion between the autophagosome and the lysosome to form the autolysosome and subsequent degradation of the cargo. (**C**) Autophagy-inducing compounds. Modulators acting through ROS-related pathways, mTORC1 inhibitors, and AMPK activators are shown.

Autophagy can be divided into five phases: initiation, nucleation, isolation membrane (IM) extension, maturation, fusion, and degradation [7]. Autophagosome formation occurs at multiple sites throughout the cytoplasm in mammals, which are located in subdomains of the endoplasmic reticulum (ER) called omegasomes [15]. Autophagosome biogenesis includes the formation of the initial IM as a flattened membrane, expanding the membrane due to the incorporation of lipids from endomembranes, bending the membrane into a sphere, and closing the membrane [6]. Finally, the autolysosome is formed due to the fusion of the autophagosome and lysosome, where the degradation of cellular components occurs (Figure 1). First discovered in yeast, autophagy is mediated by more than twenty evolutionarily conserved genes known as ATGs [14]. The major autophagic proteins involved in the mammalian process are described below and summarized in Table 1 [16].

#### 2.1.1. ULK Complex

IM formation is regulated by the ULK (Unc-51 Like Kinase) Complex (Table 1) [4]. The mammalian ULK Complex is stable and assembles independently of the cellular nutritional status [17]. In the presence of nutrients, kinase mTORC1 is active, promoting ULK1/2 and ATG13 inactivation by phosphorylation. However, mTORC1 inhibition under fasting conditions or in the presence of rapamycin allows the activation of both ULK1/2 and the phosphorylation of FIP200 and ATG13 [18]. ULK Complexes’ formation appears to occur at specific locations enriched with the enzyme phosphatidylinositol synthase (PIS). In both selective and non-selective modes, several subunits of the ULK Complex form a super-assembly that acts as a starting point for the recruitment of the remaining elements (Figure 1) [15].

#### 2.1.2. PI3K-III Complex

The activation of the ULK Complex promotes the recruitment of PI3K-III to ATG9 vesicles or a membrane compartment containing the late endosomal protein RAB11A (Ras-related protein Rab-11A) (Table 1) [19]. The primary function of PI3K-III, and, in particular, the catalytic subunit VPS34, involves the phosphorylation of phosphatidylinositol (PI) to phosphatidylinositol 3-phosphate (PI3P) in the IM (Table 1) (Figure 1). Compared to other membrane types in this cell, the autophagosomal membrane contains high levels of PI3P [4].

#### 2.1.3. ATG9 Vesicles

The membrane proteins ATG9A and ATG9B localize in 30–60 nm vesicles generated via the trans-Golgi network or clathrin-mediated endocytosis from the plasma membrane [19]. ATG9 vesicles bind to the nucleation site by interacting with ATG13 and FIP200. ATG9 is thought to exhibit scramblase activity (the bidirectional translocation of phospholipids without ATP) without apparent selectivity, transferring ATG2-transported phospholipids from the cytoplasmic to the luminal side of the IM and driving autophagosomal membrane expansion (Table 1) (Figure 1) [20].

#### 2.1.4. ATG2–WIPI Complex

The ATG2–WIPI Complex is recruited to the initiation site by the binding of WIPI to PI3P. ATG2A and ATG2B accommodate many phospholipid molecules that tunnel lipid transport from a donor compartment, e.g., the ER, plasma membrane or ERGIC (endoplasmic reticulum-Golgi intermediate compartment)-derived COP-II vesicles, to the cytoplasmic side of the IM (Table 1) (Figure 1) [18].

#### 2.1.5. ATG16L1 Complex

The ubiquitin-like protein ATG12 covalently binds ATG5 in an E1-type (ATG7) and E2-type (ATG10) pathway. This conjugate binds noncovalently to ATG16L1, forming the homodimeric ATG16L1 Complex, which, via WIPI2, engages with PI3P-enriched membranes [15]. Subsequently, the ATG16L1 Complex interacts with ATG3 to stimulate lipidation and the autophagosomal membrane localization of the LC3/GABARAP (GABA Receptor-Associated Protein) (Table 1) (Figure 1) [15].

#### 2.1.6. ATG8/LC3/GABARAP

The ATG8 gene family is expressed in many tissues and is involved in multiple cellular processes [6]. ATG8 orthologs in animal cells are divided into three subfamilies based on sequence similarity [21]. ATG8/LC3 proteins are processed by ATG4A or ATG4B protease, followed by E1 (ATG7), E2 (ATG3), and E3 (ATG16L1 Complex) enzymes, which leads to the synthesis of LC3-II by the addition of the membrane lipid phosphatidylethanolamine (PE) to LC3-I (Figure 1) [21]. Lipidated LC3-II binds to autophagosomal membranes, promoting elongation. LC3 also interacts with specific receptors with an essential role in cargo selectivity, such as NBR1, the ubiquitin-binding protein p62, NDP52, TAX1BP1, and optineurin (Table 1) [7]. Upon the completion of autophagosome formation, LC3 is released from the autophagosome outer membrane back into the cytosol via the ATG4 protease-catalyzed PE cleavage to be reused [14].

#### 2.1.7. ESCRT-III Complex

Once the desired size is reached, the next step is the closure of a small pore involving the ESCRT-III Complex [15]. Once recruited, the polymerization of multiple ESCRT-III subunits leads to the formation of filaments that facilitate edge approximation and allow autophagosome membrane fission (Table 1) [16].

#### 2.1.8. SNARE Proteins

The interaction between SNARE proteins localized in the lysosomal membrane and autophagosomes mediates the fusion between these two membranes. On the other hand, ATG4, the HOPS (Homotypic fusion and vacuole Protein Sorting) Complex, and RAB proteins regulate and mediate the contact between membranes (Table 1) [22]. Once fusion is completed, lysosomal enzymes enter the space bounded by the autophagosomal membranes, causing the inner membrane to degrade [15,23]. This is followed by the digestion of the cargo initially sequestered by the autophagosome [22].

### 2.2. Regulation and Signaling Pathways

The regulation of autophagy occurs at multiple levels and can be both positive and negative. The interplay between regulatory elements is vast; therefore, we focused on the most prominent and well-studied features.

#### 2.2.1. Post-Translational Regulation

**Figure 2:**
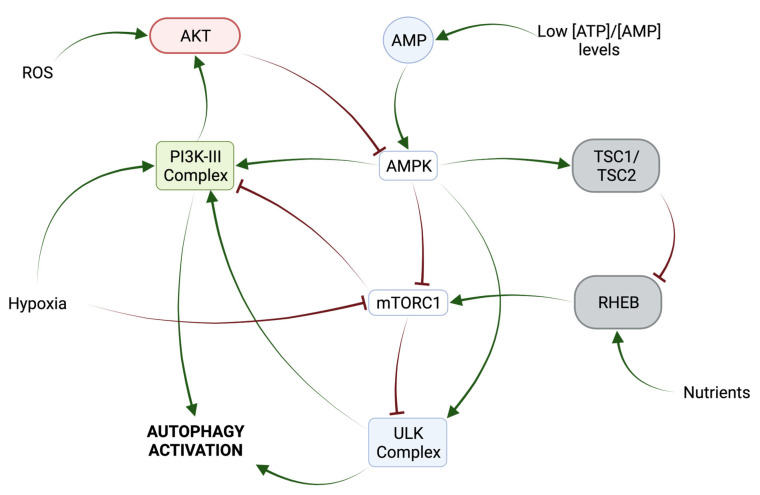
Post-translational mechanisms involved in autophagy regulation.

mTORC1 (mammalian Target of Rapamycin Complex 1) has been described as a key autophagy regulator. It is usually active in situations where the energy level is high, when growth factors are present, or when glucose and amino acids are prevalent [24]. Then, mTORC1 phosphorylates and inactivates the ULK Complex and VPS34 (Beclin1-VPS34-ATG14/UVRAG) Complexes (Figure 2) [25]. Under stress conditions, when the amino acid or ATP concentration decreases, AMPK (Adenine Monophosphate-activated Protein Kinase) is activated by a variety of mechanisms, including LKB1 (Liver Kinase B1)-mediated phosphorylation and the binding of the allosteric effector AMP [26]. Once activated, AMPK negatively regulates mTORC1 through several molecular mechanisms (Figure 2). On one hand, it phosphorylates and activates the tumor suppressor proteins TSC2/TSC1 (Tuberous–Sclerosis Complex 1/2) (Figure 2). On the other hand, AMPK inhibits the activity of RHEB (Ras Homolog Enriched in Brain), which is required for mTORC1 activity (Figure 2) [27]. In addition, AMPK negatively regulates the activity of the mTORC1 subunit RAPTOR (Regulatory-Associated Protein of mTOR) [26]. In addition, AMPK positively regulates the ULK1/2 Complex through phosphorylation and promotes the activation of PI3K-III by phosphorylating Beclin1 [25,28].

Another important regulator of autophagy is AKT kinase, which can inhibit autophagy through several effectors: by phosphorylating AMPK in a pathway involving insulin and its receptor IRS1 (Insulin Receptor Substrate 1) or by activating mTORC1 in response to increased reactive oxygen species (ROS) levels and under nutrient-rich conditions by repressing the activity of FOXO (Forkhead box O) transcription factors (Figure 2) [27]. In addition, AKT cooperates with EGFR in the inhibition of autophagy through the phosphorylation of Beclin1 in a manner independent of the mTORC1 pathway [28]. Under unperturbed conditions, Beclin1 binds to Bcl2 (B-cell lymphoma 2), modulates the PI3K Complex, and inhibits autophagy, whereas, under stress conditions, the interaction between Beclin1 and Bcl2 is disrupted by Beclin1 phosphorylation [5].

In addition, the activation of N-terminal c-Jun kinase (JNK) due to ROS leads to phosphorylation and the inactivation of Bcl2, thereby promoting autophagy [5]. HMGB1 (High Mobility Group Box 1) plays a positive role in autophagy induction during oxidate stress via its translocation from the nucleus to the cytosol and its interaction with the autophagy protein Beclin1, displacing Bcl2 [29].

#### 2.2.2. Transcriptional Regulation

Different types of stress, including metabolic, oxidative, and ER stress, influence gene transcription through multiple transcription factors, either promoting or inhibiting autophagy. For instance, during fasting or oxidative stress, the transcription factor EB (TFEB), a critical regulator of autophagy, translocates to the nucleus and promotes gene expression due to reduced mTORC1-mediated phosphorylation [30].

The versatile tumor suppressor protein p53 responds to cellular stress and exerts differential effects on autophagy based on its cellular location; nuclear p53 stimulates autophagy through gene activation, while cytoplasmic p53 hampers this process [31,32].

A central regulator, ZKSCAN3 (zinc finger with KRAB and SCAN domains 3), modulates lysosome biogenesis-related genes via shifting its location between the nucleus and cytoplasm in response to nutrient availability [33]. FXR (Farnesoid X Receptor), another transcription factor regulated by the nutritional stage, predominantly localizes in the nucleus during unperturbed conditions to repress ATG gene expression through competition with PPARα [34]. Finally, nuclear factor κB can either induce ATG gene expression, like Beclin1 or trigger p62 accumulation, linked explicitly to mitophagy [35].

#### 2.2.3. Post-Transcriptional Regulation

The regulation of autophagy involves many non-coding RNAs, such as microRNAs and lncRNAs. MicroRNAs are single-stranded non-coding RNAs (22–24 nucleotides) that bind to the 3′-UTR of their target mRNA [36,37], inhibiting mRNA translation or promoting mRNA degradation [38]. Different microRNAs regulate the elongation phase of autophagy (miR-224, miR-181a, miR-374a and miR-30a), ATG9 retrieval (miR-34a), autophagosome maturation and lysosome fusion (miR-373, miR-502 and miR-451) [36]. In addition, lncRNAs regulate ATG gene expression through their interaction with microRNA targets [30,39].

#### 2.2.4. Epigenetic Regulation

Autophagic flux is regulated by a variety of epigenetic modifications, including DNA, histone modifications (acetylation, methylation, phosphorylation, or ubiquitination), and chromatin remodeling [30,40]. AMPK is a key regulator of these epigenetic events. It directly phosphorylates histones and DNA methyltransferases [40] and has been linked to the activation of the histone deacetylase (HDAC) SIRT1: an autophagy inducer [41]. EZH2 (Enhancer of Zeste Homolog 2) also activates mTORC1 and represses autophagy by methylating histone H3 [40].

## 3. Modulation of Autophagy in OS Therapeutics

### 3.1. Role of Autophagy in Bone Homeostasis and Tumorigenesis

Bone consists of four main cell types: osteoblasts (OB), osteoclasts (OC), osteocytes (OCT), and bone lining cells. The bone marrow stroma contains mesenchymal stem cells (MSCs), from which osteosarcoma (OS), a malignant bone tumor, arises. MSCs are undifferentiated cells that have the capacity for self-renewal, proliferation, and differentiation into various cell types, including osteoblasts, which are responsible for bone formation. Normal osteogenesis, the process by which osteoblasts are formed from MSCs, is regulated by several intrinsic and extrinsic factors. Disruptions to this regulatory process, such as alterations in these factors or exposure to non-native stimuli, such as pro-inflammatory cytokines and pro-tumor agents, can lead to an imbalance between cell differentiation and proliferation, ultimately contributing to the development of a malignant phenotype in OS [42]. Multiple lines of evidence suggest that autophagy plays a crucial role in the remodeling and regeneration of bone tissue because it contributes to pre-osteoblast differentiation, the transition from osteoblasts to osteocytes, and the functioning of osteoclasts [43]. Importantly, increased autophagosome degradation plays an important role in MSCs’ differentiation into OBs by providing energy and metabolic precursors [44]. OBs, as bone-forming cells, secrete the organic matrix of bone and participate in the mineralization process. The lack of autophagy reduces osteoblast mineralization and disrupts the balance between osteoblasts and osteoclasts, leading to an overall decrease in bone mass [45]. Autophagic proteins are also required for osteoclast-directed bone resorption and the differentiation of osteoblasts into osteocytes [46]. Given the key role of autophagy in maintaining the physiological homeostasis of bone, therapeutic interventions targeting the autophagic process could allow the imbalance between differentiation and proliferation that characterizes OS to be regulated. However, it is worth noting that a general induction or reduction in autophagy may simultaneously affect both bone formation and resorption [43].

Tumor cells, including OS, are characterized by genomic alterations and invasive growth and are normally surrounded by a microenvironment with distinct biochemical and biophysical characteristics, such as hypoxia, acidosis, high interstitial fluid pressure, and extracellular matrix stiffness. In tumors, autophagy can be either promotive, suppressive, or neutral. Autophagy appears to act as a suppressor of tumorigenesis, helping to alleviate oxidative stress and genomic instability to keep cells healthy [36]. When autophagy is defective, inflammation is stimulated, and a permissive environment for tumor development is created [47]. The induction of autophagy in the primary stages of OS development could be a possible strategy for prevention or treatment [48]. However, autophagy can promote tumor spread and growth by providing nutrients and energy in the hostile microenvironment created by tumor cells and by contributing to resistance in therapy [49]. This dual role of autophagy in cancer raises the question of whether inhibiting or stimulating autophagy is better for OS treatment [50]. Either way, the pharmacological modulation of autophagy holds promise as a novel approach to improve therapeutic efficacy against OS [49]. In the subsequent sections, we provide an exhaustive examination of the studies conducted using various drugs to manipulate autophagy in the context of osteosarcoma. A comprehensive overview of compounds in the preclinical stage, demonstrating their potential to modulate osteosarcoma (OS), can be found in Table 2. Conversely, those compounds already undergoing clinical testing are detailed in Table 3.

### 3.2. Autophagy Inhibitors

Different studies have shown that drug-induced cancer cell death can be effectively increased by disrupting ATG genes and inhibiting autophagy [51]. However, it is noteworthy that conflicting results have been reported depending on the use of different inhibitors or silencing targets [49,52,53]. In addition, there are a number of considerations to be made when choosing the most appropriate treatment. For instance, autophagy inhibitors are typically combined with targeted therapy regimens to enhance the anti-tumor activity and efficacy of chemotherapeutic drugs [32]. However, the toxicity of global autophagy inhibition is an important reason to carefully consider this process as a target, as it does not specifically target tumor cells [54]. Similarly, the chronic inhibition of autophagy should be evaluated with caution due to its critical role in the homeostasis of non-tumor cells [11]. In addition, adaptation to autophagy inhibition is an issue that needs to be considered. Although clinical trials empirically testing resistance to pharmacological autophagy inhibition in patients have not yet been conducted, some published studies already show evidence of acquired resistance in patients with solid tumors treated with CQ and HCQ [55]. Future studies on this phenomenon may reveal the underlying molecular mechanisms, which could have implications for the design of appropriate combinatorial therapies [55]. Pharmacological inhibitors may be classified by the stage at which they intervene: early-stage inhibitors target elements or signaling involved in the early steps of autophagy, while late-stage inhibitors target the lysosomal function (Figure 1) [56].

#### 3.2.1. Early-Stage Inhibitors

##### PI3K Inhibitors

**Figure 3:**
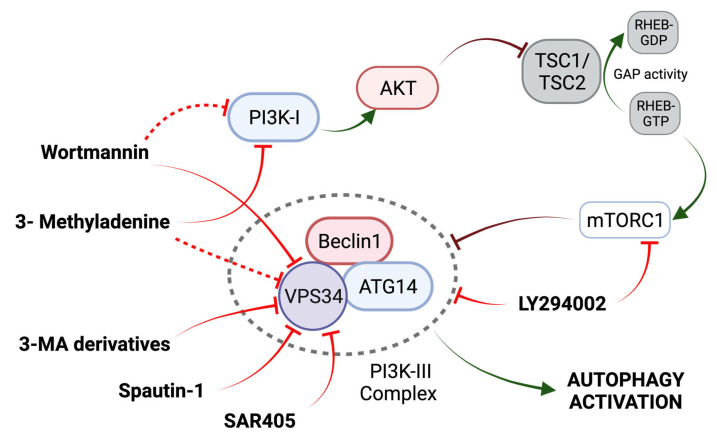
Mechanism of action of PI3K-III Inhibitors. Activation of the PI3K-I/AKT pathway leads to the activation of mTORC1 through the disruption of TSC2 GAP activity. See main text for further details. Adapted from [58].

LY294002, 3-Methyladenine (3-MA), and wortmannin are autophagic inhibitors that act through the inhibition of PI3K (Figure 3) [57]. 3-MA acts through the transient inhibition of PI3K-III (VPS34) and permanent inhibition of PI3K-I (a positive regulator of mTOR) [56]. Activated class PI3K-I leads to the activation of AKT, which inactivates the GAP activity of TSC2 for the small G protein Rheb and favors the promoter effect of Rheb (bound to GTP) to mTORC1 [58,59]. Three 3-MA derivatives with increased solubility and autophagy inhibition efficacy have been described, although their potential remains limited [60]. In OS, the use of 3-MA can significantly improve OS cell sensitivity to chemotherapeutic agents (Table 2) [61].

Wortmannin, a fungal metabolite, is a more potent autophagic inhibitor compared to 3-MA, permanently inhibiting PI3KIII (VPS34) and transiently inhibiting PI3K-I [59]. However, wortmannin also has inhibitory effects on other kinases, like mTOR [56]. Finally, the synthetic PI3K and mTOR inhibitor LY294002 increases the chemosensitivity of OS cells in combination with cisplatin (CDDP) [56].

In general, PI3K inhibitors have limited potency and act in a non-specific manner, and the characterization of their pharmacological properties is still under investigation. Some authors point out that this lack of specificity of PI3K inhibitors makes them unsuitable for a clinical setting [48]. However, the chemical modification of autophagy inhibitors, as evidenced by the development of three 3-MAs [60], could be an effective strategy in the search for improved inhibitors.

In addition to general PI3K inhibitors, there are two specific VPS34 inhibitors, Spautin-1 and SAR405 (Figure 3). Spautin-1 enhances the degradation of VPS34 complexes by ubiquitinating Beclin1, followed by proteasomal degradation [56]. Although the use of Spautin-1 in OS has only been evaluated in one study, synergistic anti-tumor effects have been observed when combined with rapamycin in other tumors [62]. On the other hand, SAR405 potently inhibits the catalytic activity of VPS34 [56]. The combination of SAR405 and celecoxib, an inhibitor of prostaglandin synthesis (a marker of poor tumor prognosis), enhanced the celecoxib-mediated suppression of cell viability in OS xenografts (Table 2) [63]. The gene silencing of ATG5 had a similar effect to that of SAR405 [63]. Thus, the inhibitory effect of these compounds is an added therapeutic value with potential in the treatment of OS.

##### ATG4B Inhibitors

**Figure 4:**
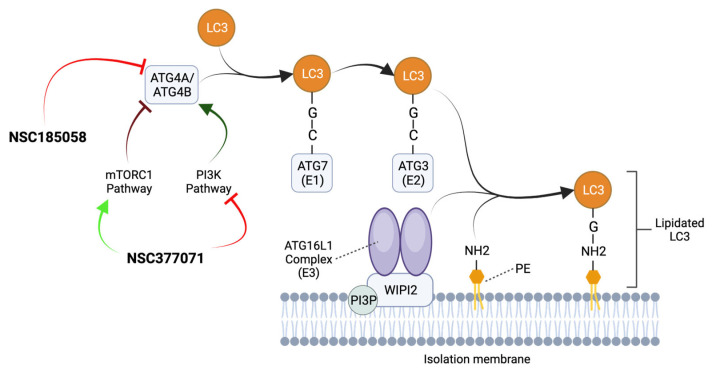
Mechanism of action of ATG4B inhibitors. ATG4A/ATG4B cleaves LC3 proteins at the C-terminal tail to expose their glycine residues. Then, ATG7 (E1), ATG3 (E2), and the ATG16L1 Complex (E3) collaborate to conjugate LC3 to the amino group (NH2) of PE present in the IM. The ATG16L1 Complex is recruited to PI3P-positive autophagic membranes by binding to a WIPI2-interacting region. The ATG4B antagonists NSC377071 and NSC185058 suppress ATG4B activity and inhibit autophagy by blocking LC3 lipidation and autophagosome formation. NSC185058 has little or no effect on mTOR or PI3K activity, while NSC377071 appears to positively affect the mTORC1 pathway and/or negatively affect the PI3K pathway. Adapted from [15,64,65].

ATG4B plays a positive role in the maintenance and growth of OS xenografts, with its absence leading to attenuated tumor growth and even death in mouse models [64]. To date, two ATG4B inhibitory compounds, NSC185058 and NSC377071, which suppressed LC3 lipidation in OS cells under starvation conditions, have been tested. NSC377071 may suppress ATG4B activity by positively regulating the mTOR pathway and/or PI3K pathway down-regulation, whereas NSC185058 has little impact on PI3K or mTOR activity (Figure 4) [64]. Both compounds may inhibit autophagy in OS cells in vivo and suppress tumor growth in preclinical models. However, the protein targets of these inhibitors remain to be defined in detail.

#### 3.2.2. Late-Stage Inhibitors

**Figure 5:**
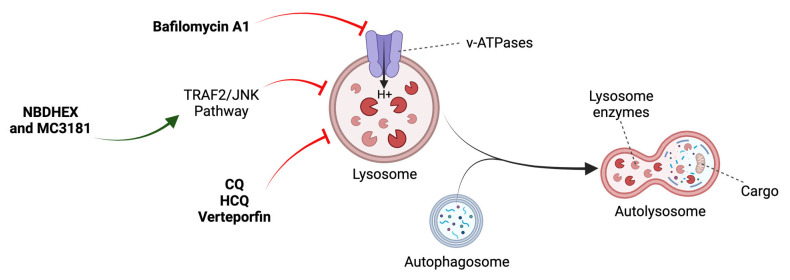
Mechanism of action of late-stage inhibitors. See main text for details. Adapted from [48].

Chloroquine (CQ) and hydroxychloroquine (HCQ) deacidify and block the fusion of autophagosomes with lysosomes (Figure 5) [50]. However, they can interfere with other critical biological processes. Different authors have suggested that their anticancer effects are independent of autophagy, simply by triggering lysosomal lysis and cell death (Table 3) [64].

Verteporfin, a drug used in the treatment of ocular degeneration, is able to disrupt autophagy at multiple levels in OS cells from the disruption of early autophagic processes, the induction of lysosomal instability, and inhibition of autophagic flux (Figure 5) [66]. Bafilomycin A1, which inhibits lysosomal acidification, has been combined with 3-MA and delphinidin, another autophagic inhibitor, and cytotoxic effects have been observed in OS cells via the accumulation of ROS and impairment of cellular protective mechanisms (Figure 5) (Table 2) [67,68].

The nitrobenzoxadiazole (NBD) derivatives NBDHEX and MC3181 trigger autophagic impairment and cell death by JNK activation (Figure 5) (Table 2) [69]. New evidence supports the negative role of JNK in autophagy in certain cellular contexts [69,70]. However, much of the scientific literature has established it as a positive regulator. These findings open the way for new treatments that exploit the potential of NBDs as anticancer agents in OS [69].

EGCG (Epigallocatechin-3-Gallate), a green tea polyphenol with anti-tumor bioactivity, was found to inhibit DOX-induced pro-survival autophagy in OS [70]. In the same study, the authors showed that EGCG could partially inhibit the self-renewal capacity of OSCs [70], providing a basis for developing drugs to target OSCs and, therefore, improving the clinical efficacy of chemotherapy (Table 2). The molecular mechanisms involved in the action of EGCG in OS require further studies. In other types of cancers, EGCG acts through multiple signaling pathways, including p38/MAPK and PI3K/AKT, to promote autophagy [71]; however, the heterogeneity of OS can pose a problem when extrapolating results. This is an important fact when considering the anti-tumor effects of EGCG and other drugs in OS.

**Table 2:** Potential autophagy modulators in OS therapy.

		**Modulators That Induce Autophagy**		
**Compound**	**Target**	**Main Findings**	**Refs.**	Stage of Clinical Development
Aloin	PI3K/AKT/mTOR	Inhibits proliferation and promotes apoptosis	[72]	Preclinical, In vitro
Andrographolide	PI3K/AKT/mTOR and JNK	Inhibits viability, induces autophagic death and reduces invasion and metastasis	[73]	Preclinical, In vitro
Baicalin	PI3Kγ	Due to the ROS and Ca2+ accumulation, it causes the loss of mitochondrial membrane potential	[74]	Preclinical, In vitro
Capsaicin	ROS-related pathways	Combined with DDP, it is able to inhibit OS cell viability and invasion	[75]	Preclinical, In vitro
Celastrol	ROS/JNK	Blocks OS cell proliferation by inducing G2/M phase arrest	[76]	Preclinical, In vitro
Cinobufagin	ROS/JNK/p38	Triggers apoptosis and autophagic cell death	[77]	Preclinical, In vitro
Curcumol	JNK	Antineoplastic effect through induction of cell apoptosis	[78]	Preclinical, In vitro
Diallyl Disulfide	PI3K/AKT/mTOR	Induction of G2/M arrest, apoptosis and autophagic death	[79]	Preclinical, In vitro
Escin	ROS/p38	Induction of autophagy and apoptosis to counteract OS proliferation	[80]	Preclinical, animal models
Estrogen Receptor β (ERβ)	mTOR	Inhibition of cell viability and mediation of cell death	[81]	Preclinical, In vitro
Germacrone	-	Anti-cancer effect, cell cycle disruption and inhibition of cell migration	[82]	Preclinical, In vitro
Ginsenoside Rg5	v/AKT/mTOR	Anti-proliferative effects by autophagy and apoptosis induction	[83]	Preclinical, In vitro
Imperatorin	PTEN/PI3K/AKT/mTOR-p21	Inhibition of tumor growth and cell cycle arrest in G0/G1	[84]	Preclinical, In vitro
CYT997(Lexibulin)	ROS-related pathways	Decreased tumor growth without obvious toxicity via the ROS and the ER stress pathways	[85]	Preclinical, In vitro
Licochalcone A/B	PI3K/AKT/mTOR	Anti-proliferative effects through induction of apoptosis and cell arrest	[86,87]	Preclinical, animal models
NVP-BEZ235	PI3K/mTOR	Synergistic enhancement of the anti-proliferative effect of CDDP	[88]	Preclinical, animal models
Parthenolide	NF-κB	Anti-cancer effects through caspase-independent autophagic cell death via ROS activation	[89]	Preclinical, In vitro
Peiminine	ROS/JNK	Suppresses proliferation and metastasis, induces cell cycle arrest and apoptosis	[90]	Preclinical, In vitro
Pelargonidin	PI3K/AKT	Anti-tumoral effects; loss of mitochondrial membrane potential and G2/M cell cycle arrest	[91]	Preclinical, animal models
PF-06409577	AMPK	Causes apoptosis and strong inhibition of cell viability and proliferation	[92]	Preclinical, In vitro
Proflavine	HIF-1α pathways	Promotes apoptosis and inhibits the growth of OS cells	[93]	Preclinical, In vitro
Quercetin	ROS-NUPR1 pathway	Induces cell death in OS cells through the induction of excessive autophagy	[94]	Preclinical, animal models
Tetrahydrocurcumin	PI3K/AKT/mTOR and p38	Induces mesenchymal–epithelial transition, suppresses angiogenesis and lung metastasis, and inhibits cell activities	[95]	Preclinical, In vitro
Triptolide	Wnt/β-Catenin	Inhibits angiogenesis and induces apoptosis	[96]	Preclinical, In vitro
Stat3-MA	PI3K	Enhances cytotoxic effects by combining with CDDP	[61,97]	Preclinical, In vitro
Bafilomycin A1	H+ ATPase	Inhibits cell proliferation, induces apoptosis	[68,98]	Preclinical, In vitro
Delphinidin	ROS-related pathways	Allows ROS to accumulate and ultimately promotes apoptotic cell death	[68]	Preclinical, In vitro
Epigallocatechin-3-gallate (EGCG)	lncRNA SOX2OT	Inhibits the tumor characteristics of OS cells and prevents tumor cells from metastasizing	[70,71]	Preclinical, In vitro
LY294002	PI3K/mTOR	Increases CDDP chemosensitivity	[61]	Preclinical, In vitro
NBDHEX and MC3181	TRAF2 and JNK	It weakens the ability of tumor cells to withstand stress conditions	[69]	Preclinical, In vitro
NSC185058 and NSC377071	ATG4B	Decreases tumor growth and size	[64]	Preclinical, In vitro
SAR405	VPS34	Enhances celecoxib-induced inhibition of cell proliferation	[63]	Preclinical, In vitro
Spautin-1	VPS34	Improves the suppression of cell proliferation and apoptosis	[62]	Preclinical, In vitro
Verteporfin	-	Induces the sensitization of OS cells and enhances cytotoxicity	[66]	Preclinical, In vitro
Wortmanine	PI3K	Enhances antitumor effects when combined with CDDP	[67,99]	Preclinical, In vitro

#### 3.2.3. Current Use of Autophagy Inhibitors in Clinical Practice

One of the primary challenges encountered in the clinical translation of autophagy inhibitors lies in their limited efficacy within animal models [54]. Nonetheless, it is essential to note that the number of compounds currently under investigation in clinical trials for cancer remains relatively modest. For instance, within the context of osteosarcoma (OS), a noteworthy clinical trial (NCT03598595) is underway, assessing the combined utilization of chloroquine (CQ) and hydroxychloroquine (HCQ) alongside gemcitabine and docetaxel, targeting patients who exhibit resistance to conventional chemotherapy regimens (as detailed in Table 3).

Among the compounds under early-stage investigation for potential osteosarcoma treatment, several have ventured into human clinical trials for alternative cancer types. For instance, LY294002 is currently undergoing phase 1 clinical trials with a focus on patients afflicted by neuroblastoma (referenced as NCT02337309) (Table 2). Conversely, both verteporfin and epigallocatechin gallate (EGCG) have advanced to phase 2 trials (NCT03033225), although it is noteworthy that verteporfin’s application is primarily geared toward the treatment of pancreatic tumors (Table 2). Meanwhile, EGCG is being assessed for its effectiveness as a protective agent for the skin of breast cancer patients who are undergoing radiotherapy (NCT02580279) (Table 2).

### 3.3. Autophagy Inducers

The depletion of critical cellular components due to excessive autophagic activity is the major cause of autophagic cell death [100]. As apoptosis is often inactivated in human cancers, the induction of autophagy may be an alternative mode of promoting cell death [51]. The main characteristics and mechanisms of action in autophagy-inducing agents used to treat OS are described below. They can be divided into three main groups: inducers related to the AMPK pathway, inducers related to the PI3K/AKT/mTOR pathway, and autophagy inducers involved in other pathways.

#### 3.3.1. mTOR Inhibitors

**Figure 6:**
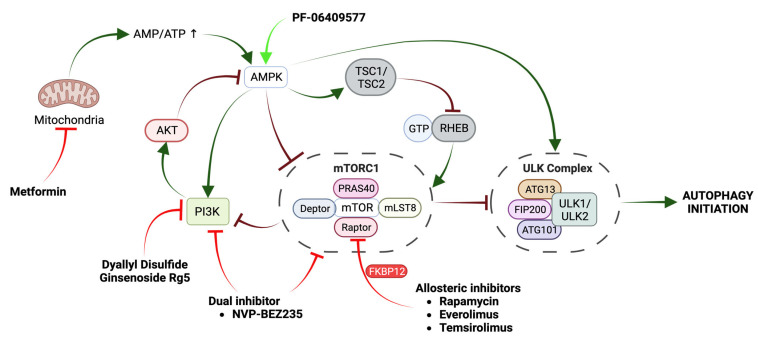
Mechanism of action of mTOR inhibitors and AMPK inducers. See main text for details. Adapted from [101,107]. Green arrows denote activation, red arrow indicates inhibition.

Rapamycin is a natural mTORC1 inhibitor that has been approved for use as an immunosuppressive agent. Rapamycin forms a complex with FKBP12 and binds to the rapamycin/FKBP12 binding domain in mTOR, resulting in RAPTOR dissociation and mTORC1 inactivation (Figure 6) [101]. Thus, the allosteric inhibition of mTORC1 activity using rapamycin can increase tumor autophagy and reduce tumor growth by inducing cell death [102]. However, results in clinical trials for the treatment of cancer have been less than promising due to its poor solubility and pharmacokinetic properties. This has led to the development of several water-soluble analogs, such as temsirolimus (CCI-779) (Table 2) and everolimus (RAD001) (Figure 6) (Table 3). Interestingly, the combination of rapamycin or its derivatives with an autophagy inhibitor enhances their cytotoxic effects by accumulating autophagosomes to accelerate tumor cell death in vitro and in vivo in human cancer xenograft models (Table 2) [103,104].

Dual PI3K-mTOR inhibitors, such as NVP-BEZ235, ATP analog mTOR inhibitors, such as AZD8055 or WYE132, and AKT inhibitors have been developed to provide a more effective blockade of the mTOR pathway [51,105]. Currently, dual ATP-competitive inhibitors of mTOR or AKT are currently being investigated as potential drugs for the treatment of non-OS tumors [105]. In addition, a new generation of mTOR inhibitors, such as RapaLink, is being investigated for the treatment of cancer, although its relevance to the treatment of OS has not yet been assessed [105].

Despite the interest in mTOR as a therapeutic target, this signaling pathway is also critical for numerous cellular processes, including metabolism. Blocking mTOR activity can, therefore, lead to additional clinical side effects that are unrelated to autophagy induction. These include hyperglycaemia, hyperlipidaemia, pneumonitis, stomatitis and hepatotoxicity [50,105]. The mechanisms leading to these toxicities need to be analyzed, as they could limit the approval of these agents.

In addition, a wide variety of natural products that stimulate autophagy by interfering with the PI3K/AKT/mTOR pathway in OS cells are described in Table 2 [106].

#### 3.3.2. AMPK Inhibitors

Metformin is a drug used to treat type 2 diabetes, promoting autophagy by indirectly activating AMPK (Figure 6). The inhibition of Mitochondrial Respiratory Chain Complex I enhances the AMP/ATP ratio, promoting AMPK activation and the subsequent inactivation of the mTORC1 Complex [48]. Numerous studies have revealed that metformin may inhibit proliferation and induce cell death in many cancers, including OS [108]. Thus, metformin correlates with a reduced risk of cancer incidence in diabetic patients, suggesting a preventive role that could also be seen in OS (Table 3) [109]. However, its use is limited due to poor bioavailability, off-target toxicities, and, often, low efficacy [92]. Recently, the role of PF-06409577, a synthetic drug that selectively binds to AMPK subunits, has been studied in OS cell lines (Figure 6) [92]. The effects of PF-06409577 are significantly more potent than traditional activators, such as metformin, representing significant advances in this field (Table 2). In addition to metformin, other drugs, such as AICAR, CRO15, α-hederin, phenformin, 2-deoxy-D-glucose (2DG), A-769662 or salicylate, stimulate autophagy through direct or indirect AMPK activation [102,107]. However, their specific mechanisms of action and possible adverse effects have not yet been fully elucidated and evaluated. It should also be noted that their potential activity has only been assessed in models of neoplasia, such as melanoma or colorectal cancer [110], and not in OS.

#### 3.3.3. Autophagy Inducers Involved in Other Pathways

Numerous natural compounds stand out for their ability to influence multiple pathways related to promoting autophagy in OS, such as the ROS/JNK pathway or the HIF-1α-related pathway (Figure 1). Also, the effect of microtubule-targeting compounds that are capable of perturbing tumor cell architecture and inducing autophagic cell death has been explored [85]. Taken together, these studies expand our knowledge of the benefits and clinical use of these compounds and lay the groundwork for their use as drugs for the treatment of OS (Table 2).

Although their anticancer properties are well known, the exact mechanisms of action require further study [111]. They also suffer from low bioavailability, which could be overcome by chemically modifying or nano-encapsulating them.

#### 3.3.4. Current Use of Autophagy Inducers in Clinical Practice

A greater number of inducing modulators are being used in clinical trials in comparison to autophagy inhibitors. In the context of osteosarcoma (OS) treatment, notable developments include Gemcitabine, which is currently undergoing phase 1 trials in combination with Selinexor (NCT04595994) and phase 2 trials in conjunction with Rapamycin (NCT02429973). Furthermore, Metformin is in the midst of phase 2 trials, positioned as a cytostatic agent for OS (NCT04758000), while Everolimus is undergoing an examination in combination with Sorafenib (NCT01804374). Equally noteworthy are the ongoing clinical trials involving INK-128 (NCT02987959) and Panobinostat (NCT04897880), both evaluated as standalone agents without concomitant drug interventions, as delineated in Table 3.

Among those that are in their preclinical phases for OS treatment, Escin has already been approved for use in thyroid cancer [112]. Additionally, several compounds have progressed into phase 1 clinical trials, demonstrating their potential in various cancer contexts. For instance, Tripolide is currently undergoing evaluation in combination with Osimertinib for the treatment of lung cancer (NCT05166616). NVP-BEZ235 is being investigated alongside Rapamycin for its potential in leukemia therapy (NCT01756118). Likewise, Licochalcone A is being explored in conjunction with Paclitaxel as a treatment strategy for oral cancer (NCT03292822). Quercetin, Pelargonidin, and CYT997 are in phase 2 trials for prostate cancer (NCT03493997), breast cancer (NCT01936064), and myeloma treatment (NCT00664378), respectively. Finally, Andrographolide is in a phase 3 trial as a palliative treatment for patients with esophageal cancer (NCT04196075) (Table 2).

### 3.4. MicroRNA Modulation

MicroRNA is involved in tumorigenesis, metastasis, and the survival of OS cells [113]. As autophagy is also regulated by various microRNAs, they are being investigated as potential targets to modulate tumor development. Two strategies are currently under study for microRNA-based therapeutics: microRNA mimetic compounds (e.g., microRNA restoration, replacement, or overexpression) and antagomiR (e.g., microRNA inhibition and downregulation) [38]. The most important microRNAs used to modulate autophagy with anti-tumor effects in OS are reviewed in Table 4.

**Table 3:** Autophagy modulators clinically tested in OS.

**Modulators That Induce Autophagy**				
**Compound**	**Target**	**Main Findings**	**Refs.**	**Stage of Clinical Development**
Everolimus (RAD001)	mTOR	The combination of sorafenib and everolimus resulted in enhanced anti-proliferative and pro-apoptotic effects, impaired tumour growth, enhanced anti-angiogenesis and reduced migratory and metastatic potential	[114]	Phase 2 (NCT01804374)
Gemcitabine	-	Combination treatment with Rapamycin improved the suppression of tumour growth and metastasis, validating their use as monotherapies	[115]	Phase 1 (NCT04595994)
INK-128	mTORC1/2	Strong cytotoxic and pro-apoptotic activities	[116]	Phase 2 (NCT02987959)
Metformin	AMPK/mTOR	Suppressed the self-renewal ability and tumourigenicity of OSCs via G0/G1 phase arrest and ROS-mediated apoptosis and autophagy	[117]	Phase 2 (NCT04758000)
	ROS/JNK	Cell cycle arrest, apoptosis and enhanced cytotoxicity when combined with CQ	[118]	
Rapamycin	mTOR	Enhanced their anti-tumour effects when combined with chemotherapeutic agents, as well as autophagy inducers or inhibitors	[62,115,119]	Phase 2 (NCT02429973)
Panobinostat	PI3K	Induced Benclin1 expression and promoted cell death by apoptosis	[120]	Phase 2 (NCT04897880)
CQ and HCQ	Lysosome	Enhanced the cytotoxic effects of chemotherapeutic agents and autophagy inducers	[57,118,121]	Phase 2 (NCT03598595)

One of the main problems with microRNA-based therapeutics is their efficient and precise delivery to sites of interest. Current methods already have some shortcomings in this regard, such as low transfection efficiency, rapid degradation, and abnormal accumulation in non-specific tissues and organs [1]. The use of liposomes to avoid microRNA degradation or the use of viral vectors have been postulated as noteworthy alternatives [122].

**Table 4:** Modulation of autophagy in OS by microRNAs.

MicroRNA	Target	Effect on Autophagy	Main Findings	Refs.
miR-17-5p	PTEN	Inhibition	Upregulated in OS: its suppression leads to an increase in autophagy and a decrease in OS cell viability	[123]
miR-19	p38α	Inhibition	Upregulated in OS cells: miR-19 inhibitor reduces cell proliferation, invasion, migration and EMT by promoting autophagy	[124]
miR-29a-3p	PI3K/AKT/FOXO3	Promotion	Down-regulated in OS; overexpression promotes autophagy and suppresses OS progression	[125]
miR-145	HDAC4	Promotion	Down-regulated in OS; overexpression significantly attenuates proliferation and induces apoptosis and autophagy	[126,127]
miR-506-3p	SPHK1	Inhibition	EMT is stimulated, and the invasiveness of OS cells is reduced after the transfection of the miR-506-3p mimetic	[128]

### 3.5. Modulators Associated with Epigenetic Modifications

OS should be considered a differentiation disease preventing mesenchyme stem cells’ differentiation into osteoblasts [129]. Epigenetic regulators can control histone modification and affect autophagic flux. Recent studies have demonstrated that histone deacetylase inhibitors (HDACIs), including SAHA, TSA, and SB, can trigger autophagy in human cancer cells: an effect that has been linked to their anticancer properties [121,130]. Specifically, TSA is able to inhibit the mTOR pathway and increase the transcriptional activity of FOXO1, enhancing its effects when combined with CQ or HCQ [121]. SAHA and SB significantly inhibit OS cell growth and promote cell cycle arrest [130].

EZH2 alters gene expression and inhibits autophagy by catalyzing the trimethylation of histone H3 [129]. Therefore, the use of EZH2 inhibitors could induce autophagy in OS cells. GSK343 is an EZH2 inhibitor that can be effective when compromising viability in OS cells [129].

## 4. Role of Autophagy in OS Resistance

Cancer cells can use several mechanisms, including autophagy, to evade or counteract cytotoxic stimuli induced by cancer therapy [122]. Specifically, autophagy generally performs a pro-survival role and can be induced after treatment, allowing them to escape apoptosis and maintain a dormant state that contributes to recurrence and metastasis [47]. A total of 35–45% of patients with OS are insensitive to chemotherapy drugs, leading to treatment failure and poor prognosis [67]. In this regard, the limited efficacy of conventional treatments based on cytotoxic agents such as DOX, CDDP, and MTX has been certified [131]. The high level of heterogeneity observed in OS is challenging for proper therapy, making it difficult to identify reliable biomarkers [122].

### 4.1. Chemoresistance

Tumor-resistance chemotherapy based on MTX, DOX, and CDDP has no alternative treatment [132]. In OS, accumulating evidence suggests that autophagy is critical for chemoresistance by promoting drug resistance or increasing drug sensitivity [67], suggesting that the role of autophagy in chemoresistance might depend on the differential regulation of signaling and/or chemotherapeutic strategies [133]. Introducing new drugs or techniques that are capable of overcoming chemoresistance and inhibiting metastasis could further improve survival in patients with OS [132].

#### 4.1.1. High Mobility Group Box 1 (HMGB1)

HMGB1, an essential protein in the bone microenvironment, is involved in an important mechanism of tumor cell chemoresistance [134,135]. HMGB1 binds to Beclin1, facilitating dissociation from Bcl-2 and stimulating autophagy [135]. In OS cell lines, the chemotherapeutic agents CDDP, DOX, and MTX have been shown to significantly upregulate HMGB1 expression [135]. The RNA interference (siRNA)-mediated suppression of HMGB1 results in decreased autophagy and increased sensitivity to chemotherapeutic agents [135]. The depletion of ULK1 or FIP200 abolished the HMGB1–Beclin1 interaction and increased sensitivity to anticancer drug-induced apoptosis [136], suggesting that HMGB1 could be a strategic target in OS.

#### 4.1.2. HSP90

HSP90 (Heat Shock Protein of 90 kDa) plays a key role in the regulation of autophagy, promoting autophagy and inhibiting apoptosis in response to chemotherapeutic agents [137]. CDDP, DOX, and MTX can induce an increase in HSP90 expression in OS cells [137], and this suppression of HSP90 by siRNA decreases autophagic protection in response to chemotherapy [137]. Furthermore, geldanamycin, an HSP90 inhibitor, can inhibit cell proliferation by blocking the AKT/mTOR pathway [138]. This evidence suggests that both silencing and using HSP90 inhibitors can increase the chemosensitivity of OS cells, making it a therapeutic target.

#### 4.1.3. Glial Cell Line-Derived Neurotrophic Factor (GDNF) Receptor α-1 (GFR α-1)

Glial cell line-derived neurotrophic factor (GDNF) receptor α-1 (GFR α-1) has been linked to OS cell progression and metastasis through its contribution to the development of autophagy-mediated chemoresistance [139]. CDDP, the main chemotherapeutic drug used to treat OS, induces the NFκB-dependent expression of GFR α-1, which activates autophagy via the AMPK pathway and significantly suppresses apoptosis [139]. However, the other chemotherapeutic drugs used in chemotherapy against OS, DOX, and MTX did not induce GFR α-1 expression, suggesting different signaling mechanisms [133,139]. In conclusion, GFR α-1 may act as a target for chemoresistance prevention in OS, although the signaling pathways mediating its activation remain to be elucidated [133].

#### 4.1.4. MicroRNAs and LncRNAs

In recent years, research has validated the involvement of numerous oncogenic or tumor-suppressor microRNAs in chemotherapeutic sensitivity through different mechanisms, such as autophagy [134]. Thus, the deregulation of multiple microRNAs involved in autophagy has been linked to both chemotherapy resistance and an increase in cellular chemosensitivity in OS [113]. Many of these microRNAs are negatively regulated in OS cells (Table 5). The successful inhibition of proliferation and autophagy in OS and enhanced chemosensitivity have been demonstrated by the increased expression of miR-22 expression [140,141], miR-30a [142], miR-101 [143], miR-143 [144], miR-199a-5p [145] and miR-410 [131]. In contrast to the microRNAs mentioned above, miR-193b increases chemosensitivity by promoting autophagy and subsequent autophagic cell death [146]. Collectively, these microRNAs can serve as strategic targets to overcome chemoresistance in OS therapy. Therefore, understanding the role of miR-140-5p and miR-155 in promoting autophagy provides a different viewpoint regarding the microRNAs mentioned above, such that targeting them to suppress their expression is an attractive alternative strategy (Table 5) [147,148].

Significantly, several lncRNAs are upregulated in OS cells through microRNA inhibition [134]. LncRNAs primarily act as “sponges” for specific complementary microRNAs, binding to them and inhibiting their regulatory functions, allowing free translation of target mRNAs [113]. A list of lncRNAs that influence OS chemoresistance through the regulation of different microRNAs is provided in Table 5. The identification of their role in both the autophagy and tumor phenotype offers a novel perspective in the treatment of OS, such that a strategy based on stabilizing their levels would be able to improve chemosensitivity in OS.

Involving microRNAs and lncRNAs in chemotherapy-induced autophagy may provide new approaches to developing new drugs or antineoplastic strategies [113]. However, studies may be limited by inter-individual variation and a lack of knowledge of the pathways involved. Another critical aspect of using microRNAs or lncRNAs is the optimization of both efficacy and the mode of delivery, which requires further work.

### 4.2. Radioresistance

Aberrant autophagy activation is involved in radioresistance: a known hallmark of OS [113]. However, it is important to note that autophagy has a dual role in radioresistance, as it can both prevent and induce cell death. As a result, researchers have increasingly focused on searching for clinically effective radiosensitizers [5].

First, radioresistance in OS has been associated with hypoxia, which is common in the tumor microenvironment. The main mechanism by which irradiation causes cellular death is the generation of free radicals that combine with O_2_ to form DNA-damaging ROS [156]: a process that is compromised under hypoxic conditions, leading to resistance. However, recent studies have also proposed that hypoxia promotes the activation of autophagy through HIF-1α overexpression to confer radioresistance to OS cells via ROS scavenging [156]. Inhibiting HIF-1α using siRNA or chetomine significantly reduced hypoxia-induced radioresistance [156,157]. Directly inhibiting autophagy using 3-MA also increased radiation-induced cell death [113]. By contrast, NRF2 (Nuclear factor erythroid 2-related factor 2) was upregulated in response to radiation, translocating to the nucleus where it promoted the expression of autophagy genes [158]. Thus, the inhibition of NRF2 decreased autophagy, enhancing the efficiency of radiation to kill OS cells [158].

The literature has shown little therapeutic efficacy of LET gamma rays when treating OS and the increased risk of lung metastases [159]. Efforts have been made to develop a more effective method for treating OS by using high-energy LET (Neutron) radiation. Treatment with high LET radiation results in an increased number of apoptotic OS cells due to the increased levels of autophagy and cell death [159]. These underlying mechanisms involve the inhibition of AKT phosphorylation and inactivation of mTOR [160]. Thus, high LET radiation therapy has a more significant therapeutic benefit by increasing cellular sensitivity, which may be helpful in the treatment of cases where OS has become resistant to low LET radiation.

### 4.3. Relationship with Osteosarcoma Stem Cells

Several studies support the idea that OSCs, a group of microenvironmental cells that aberrantly mature into OS cells, increase autophagic activity compared to normal OS cells [122,161]. Autophagy enables OSCs to survive in a quiescent state for extended periods of time (metastatic latency), protects them from chemotherapeutic stress, and maintains their characteristics [162]. OSC survival can result in recurrence, which is a main determinant of OS mortality. Furthermore, OSCs are a driver of tumor heterogeneity, resulting in sub-populations of OS cells with different survival mechanisms [113]. In order to improve current therapies, it is essential to characterize OSCs and the role that autophagy plays in them.

Thioridazine can stimulate and modify autophagy in OSCs, leading to the autolysis of these cells [161]. In addition, the superior tumorigenicity and chemoresistance of OSC cells compared to normal cells have been shown to be abolished by inhibiting autophagy with CQ [163]. More recently, the role of calpain-6, which is involved in the organization of the actin cytoskeleton and whose expression is abnormally increased in bone tumors, has been investigated [164]. It was found that calpain-6 promoted autophagy to maintain CSOs under hypoxic conditions; therefore, its removal blocked tumor development [164]. Other studies support reducing the self-renewal capacity of CSOs using the autophagy-inhibiting drugs EGCG and metformin [70,117].

## 5. Conclusions and Future Directions

Autophagy, a dynamic and highly regulated process, holds fundamental importance for comprehending the mechanisms underlying the emergence, development, and therapeutic resistance of osteosarcoma (OS). Dysregulated autophagy has been observed to contribute to OS pathogenesis, suggesting its modulation as a promising therapeutic approach. Several natural and synthetic compounds capable of influencing autophagy have been identified, exhibiting a potential to reduce OS cell viability and proliferation. Combining autophagy modulators with siRNA targeting autophagic machinery and conventional chemotherapeutic agents has demonstrated improved efficacy over individual treatments, providing a viable strategy to combat OS chemoresistance. Nonetheless, significant gaps in our understanding of autophagic processes remain, necessitating further research to bridge this knowledge gap and address the challenges of developing effective and safe autophagy modulators for OS treatment. The complexity of OS heterogeneity presents an additional obstacle to standardizing treatment with autophagy modulators and warrants continued investigations to optimize clinical outcomes.

## Figures and Tables

**Table 1:** Central machinery involved in autophagosome biogenesis [16].

System/Complex	Components	Function
ULK Complex	ULK1/ULK2, ATG13, FIP200/RB1CC1 and ATG101/C12orf44	Recruitment of ATG proteins to the site of autophagosome formation, membrane curvature and lipid vesicle attachment
PI3K-III Complex	VPS34, VPS15, Beclin1 and ATG14/UVRAG	Lipid binding, membrane curvature and the autophagosome–lysosome fusion process
ATG9 Vesicles	ATG9L1/ATG9L2	Interacts with the ATG2–WIPI Complex and mediates IM elongation through lipid transport from the cytoplasmic leaflet to the luminal leaflet
ATG2–WIPI Complex	WIPI1-4 and ATG2A/ATG2B	Binds to PI3P in the IM, participates in ATG9 recruitment and mediates lipid transport from a donor compartment (ER) to the cytoplasmic side of the IM
ATG16L1 Complex	ATG12, ATG5 (E3), ATG16L1, ATG7 (E1) and ATG10 (E2).	Conjugation of LC3/GABARAP with phosphatidylethanolamine (PE)
ATG8/LC3/GABARAP	LC3/GABARAP, ATG7 (E1), ATG3 (E2) and ATG4	Contributes to IM elongation and recognizes specific loads
ESCRT-III Complex	-	Responsible for autophagosome pore closure
SNARE Proteins	STX17, SNAP29 and VAMP8	Mediates fusion between the autophagosome and lysosome membrane

**Table 5:** MicroRNAs and lncRNAs involved in treatment resistance in OS.

MicroRNA	Target	Effect on Autophagy	Main Findings	Refs.
CTA	miR-210	Inhibition	Down-regulated in DOX-resistant OS cells: overexpression reduced autophagy and DOX resistance through the competitive binding of miR-210	[149]
DICER1-AS1	miR-30b	Promotion	Upregulated: removal inhibited tumor growth	[150]
FGD5-AS1	miR-154-5p	Promotion	Upregulated in OS cells and improved resistance to DOX treatment by sponging miR-154-5p: its deletion improved the sensitivity of OS cells	[39]
miR-101	LC3-II and ATG4	Inhibition	Blocks autophagy during chemotherapy and improves chemosensitivity to DOX	[143]
miR-140-5p	IP3k2 and HMGN5	Promotion	Promotes resistance to DOX, CDDP and MTX by inducing cytoprotective autophagy	[147,151]
miR-143	LC3-II and ATG2B	Inhibition	Down-regulated in OS: its enforced expression reversed DOX chemoresistance	[144]
miR-155	ATG5, LC3-II/LC3-I	Promotion	Upregulated after DOX or CDDP treatment: this promoted treatment resistance	[148]
miR-193b	FEN1	Promotion	Down-regulated in OS cells: overexpression increases chemosensitivity to CDDP	[146]
miR-199a-5p	Beclin1	Inhibition	Down-regulated in OS cells: restoring their levels promoted CDDP-induced cell inhibition	[145]
miR-30a	Beclin1	Inhibition	Down-regulated during chemotherapy: the induction of its expression reduced DOX chemoresistance	[142]
miR-410	ATG16L1	Inhibition	Down-regulated: its overexpression enhanced chemosensitivity to DOX and CDDP	[131]
miR-22	Beclin1, LC3 and ATG5	Inhibition	CDDP-induced cell proliferation was decreased in cells transfected with miR-22	[37]
	HMGB1	Inhibition	Upregulated during chemotherapy: HMGB1-promoted autophagy was inhibited	[141]
SNHG15	miR-141 and miR-381-3p	Promotion	Upregulated in DOX-resistant cells: its suppression increased DOX chemosensitivity	[152,153]
SNHG16	miR-16	Promotion	Upregulated in OS: this improved CDDP chemoresistance	[154]
Sox2OT-V7	miR-22 and miR-142	Promotion	Upregulated after DOX treatment: its silencing or inhibition enhanced the cytotoxic effects of DOX	[70,155]
